# The Influence of Vaginal, Intestinal, and Tumor Tissue Microbiota on Selected Malignant Tumors in Women

**DOI:** 10.3390/ijms27156636

**Published:** 2026-07-25

**Authors:** Anna Markowska, Hubert Wolski, Mateusz de Mezer

**Affiliations:** 1 Department of Perinatology, Gynecological Obstetric Clinical Hospital, Poznan University of Medical Sciences, Polna 33 Str., 60-535 Poznan, Poland; amarkowska@ump.edu.pl; 2 Medical Institute, University of Applied Sciences in Nowy Targ, Kokoszków 71 Str., 34-400 Nowy Targ, Poland; hubert.wolski@ans-nt.edu.pl; 3 Department of Immunobiology, Poznan University of Medical Sciences, Rokietnicka 8 Str., 60-806 Poznan, Poland

**Keywords:** microbiota, microbiome, cervical cancer, endometrial cancer, ovarian cancer, breast cancer, estrobolome, dysbiosis, HPV, immunotherapy

## Abstract

Gynecological malignancies and breast cancer impose substantial health and economic burdens. This review examines how local and systemic microbiota may affect epithelial integrity, inflammation, estrogen metabolism, and immunity. The vaginal ecosystem is the most extensively studied female microbial niche. Cervical cancer serves as the most illustrative clinical example: loss of stable *Lactobacillus crispatus* dominance and increased prevalence of anaerobic bacteria (anaerobic dysbiosis) are associated with persistent HPV infection, which directly elevates the risk of cervical precancerous lesions. The estrobolome is particularly relevant in endometrial cancer, where intestinal bacterial beta-glucuronidase activity may increase estrogen reabsorption, particularly in obesity and metabolic disease. In ovarian cancer, microbiota is being studied as a possible risk modifier in BRCA1 carriers, but the evidence remains exploratory. In breast cancer, intratumoral bacteria may shape the immune microenvironment, particularly in triple-negative disease. The primary limitation of current research is methodological heterogeneity. Low-biomass samples, such as those from the ovary or endometrium, are highly susceptible to technical contamination. Most studies are cross-sectional and cannot establish causality. Current evidence supports microbiota as a modifier, not a standalone marker or a substitute for standard diagnosis and treatment. Its most plausible near-term role is in multiparameter risk or response models, pending standardized prospective validation.

## 1. Introduction

Gynecological malignancies and breast cancer impose a significant health and economic burden. According to GLOBOCAN 2022 data, cervical cancer, endometrial cancer, ovarian cancer, and breast cancer account for millions of new cases and a significant proportion of cancer deaths in women. From a clinical perspective, these diseases are not homogeneous: they differ in etiology, course, treatment sensitivity, and prognosis. However, what remains common is that their development depends not only on genetic changes in cancer cells but also on the tissue environment, immunity, metabolism, hormones, and exposure to external factors [1].

In this context, microbiota is no longer viewed as a passive collection of microorganisms. It may regulate epithelial integrity, local and systemic inflammation, estrogen metabolism, immune activity, angiogenesis, and response to treatment [2,3,4,5,6].

Vaginal microbiota is the most thoroughly studied ecosystem of the female reproductive tract. In many women of reproductive age, bacteria of the genus Lactobacillus predominate, particularly *L. crispatus*, *L. gasseri*, *L. jensenii*, or *L. iners*. The Community State Types classification proposed by Ravel et al. remains a useful reference point, although it is now known that the name of the bacterial genus alone is insufficient to assess ecosystem function [7]. A profile dominated by *L. crispatus* is typically associated with low pH and environmental stability, while *L. iners* is more ambiguous and can occur both in healthy women and in transitional states between eubiosis and dysbiosis [7,8]. Dysbiosis is not simply infection but loss of ecosystem functions that maintain acidity, barrier integrity, microbial competition, and inflammatory control. The resulting receptor activation, cytokine release, oxidative stress, and matrix remodeling may sustain lesions initiated by HPV or other established risk factors [2,5,6].

The second axis is the gut microbiota. It influences estrogen-sensitive tissues through bacterial metabolites, short-chain fatty acids, bile acid metabolism, the intestinal barrier, systemic immunity, and the estrobolome. This concept is notably significant in endometrial cancer and some breast cancers, as the activity of bacterial beta-glucuronidases can increase the pool of estrogens returning to circulation [9,10].

However, the proper proportions must be maintained. In the literature on microbiomes, it is easy to shift from co-occurrence to language suggesting causation. This is particularly risky in low-biomass samples such as endometrium, fallopian tube, ovary, or tumor tissue. Minor contamination from reagents, skin, the cervical canal, or the operating room can affect sequencing results. Therefore, interpretation should distinguish between reproducible clinical observations, plausible mechanistic hypotheses, and speculation requiring further validation [11,12,13,14,15] (Table 1).

## 2. Nature and Purpose of the Work

This article is a narrative review. Its objective is not to compile all reports identifying differences in microbiota composition, but to synthesize data on four cancers of particular relevance to women’s health: cervical cancer, endometrial cancer, ovarian cancer, and breast cancer. Although breast cancer is not strictly classified as a gynecological malignancy, its inclusion enables a more comprehensive understanding of the estrobolome, intratumoral microbiome, and treatment response—mechanisms that are shared across gynecological oncology.

## 3. Biological Mechanisms Linking Microbiota to Carcinogenesis

**Figure 1:**
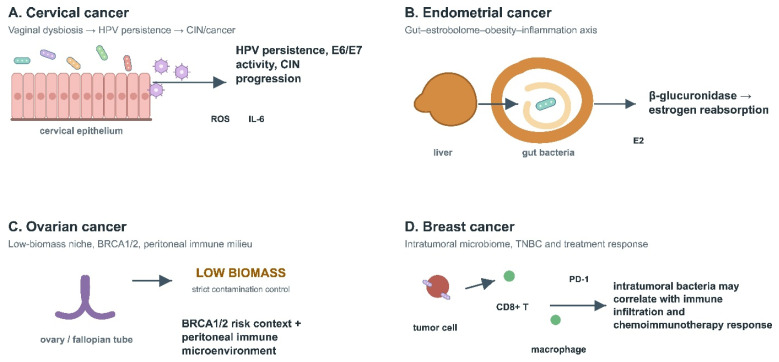
Organ-specific microbiota axes in female malignancies. The diagram summarizes the main microbiota-related axes relevant to cervical, endometrial, ovarian, and breast cancer. Cervicovaginal dysbiosis, gut-derived metabolites, estrobolome activity, and intratumoral bacteria may affect barrier integrity, inflammatory signaling, hormone metabolism, immune contexture, and treatment response. These mechanisms are organ-dependent and require cautious interpretation, particularly in low-biomass tissues.

These mechanisms do not carry equal evidential weight. Human associations are strongest for cervicovaginal dysbiosis and HPV persistence, whereas many intratumoral and ovarian mechanisms remain preclinical or hypothesis-generating. They can be organized around barrier damage, chronic inflammation, and metabolic change (Figure 1). In a healthy ecosystem, microbiota help maintain a stable environment. In the vagina, this means low pH, lactic acid production, and limited growth of anaerobic bacteria. Following a shift toward dysbiosis, the epithelium is more frequently exposed to bacterial products recognized by innate immune receptors. This results in increased NF-κB activity and the production of IL-1β, IL-6, IL-8, TNF, and reactive oxygen species. The persistence of this stimulus promotes the survival of damaged cells, angiogenesis, and immunosuppression [2,5,6].

Some studies also describe activation of COX-2-related pathways, increased expression of antiapoptotic factors, and remodeling of the extracellular matrix. These are biologically plausible mechanisms, but their significance depends on tumor location, tumor type, or the duration of the inflammatory stimulus. A short-term change in the microbiota following antibiotic therapy should be interpreted differently from chronic dysbiosis with persistent biofilm and elevated pH [5,6].

The second mechanism concerns antitumor immunity. The microbiota can influence the recruitment of dendritic cells, macrophages, T lymphocytes, and NK cells, as well as the balance between inflammation and tolerance. This is particularly important in cervical cancer, as an effective antiviral response determines the elimination or persistence of the HPV virus. Dysbiosis does not replace the action of oncoproteins E6 and E7, but may create an environment in which infected cells evade immune surveillance for longer [8,16,17,18,19,20,21,22]. Outside cervical cancer, much of this immune evidence derives from animal, pan-cancer, or extrapolated immuno-oncology studies.

The third mechanism involves metabolism. The estrobolome, a set of gut microbiome genes encoding proteins that metabolize estrogens, links the microbiota to estrogen-sensitive tumors. Bacterial beta-glucuronidases deconjugate estrogens excreted in the bile, increasing their reabsorption and bioavailability. This is a biologically plausible link between the gut, obesity, metabolic disorders, and estrogen exposure. However, this is not a simple causal relationship; it requires interpretation in the context of BMI, menopause, diet, medications, and the patient’s hormonal status [9,10].

The intratumoral microbiome is a separate issue. Multi-cancer studies have shown that bacteria can be detected within tumors, including within tumor and immune cells [13,14]. In breast cancer, pancreatic cancer, and other cancers, attempts are being made to correlate these observations with response to chemotherapy and immunotherapy [15,23,24,25]. This approach is promising but methodologically challenging. A small number of bacteria from outside the sample can produce a falsely convincing result. For this reason, tumor microbiome studies must be evaluated alongside negative controls, sample collection methods, and bioinformatic contamination filtering. Detection of bacterial DNA or structures alone does not establish functional activity or causality.

## 4. Cervical Cancer

**Figure 2:**
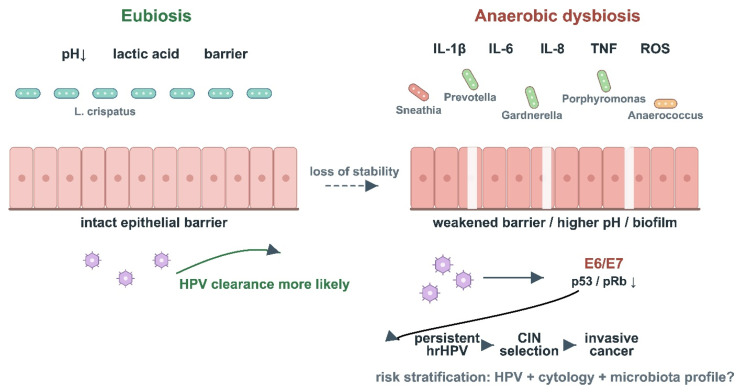
Cervicovaginal dysbiosis, HPV persistence, and cervical carcinogenesis. Loss of stable *Lactobacillus crispatus* dominance and expansion of anaerobic taxa may increase vaginal pH, weaken epithelial barrier function, promote cytokine production and oxidative stress, and alter local antiviral immunity. These changes do not replace the oncogenic effects of high-risk HPV, but may create a microenvironment that facilitates viral persistence, CIN progression, and immune escape.

Cervical cancer is the clearest example of a cancer in which the microbiota can modify the course of a process initiated by a well-defined etiological factor. In 2022, over 661,000 new cases and over 348,000 deaths due to this cancer were reported [1]. The main risk factor is persistent high-risk HPV infection, especially with types 16 and 18. Most HPV infections resolve spontaneously; therefore, factors that promote viral persistence and the progression of cervical intraepithelial lesions are of particular importance [21,22]. Oncoproteins E6 and E7 disrupt p53 and pRb function, deregulate the cell cycle, exacerbate genomic instability, and facilitate the development of CIN lesions [21]. The microbiota does not replace this mechanism, but it can alter the conditions under which HPV infection persists or is eliminated (Figure 2). Meta-analyses and systematic reviews repeat the pattern: in women with persistent HPV, high-grade CIN or cervical cancer, a stable dominance of Lactobacillus is less frequently observed, and an environment richer in anaerobic bacteria, including *Sneathia*, *Prevotella*, *Porphyromonas*, *Anaerococcus*, *Peptostreptococcus* and *Gardnerella*, is more frequently observed [16,20,26].

*L. crispatus* has attracted the most attention. Its predominance is usually associated with eubiosis, lactic acid production, and lower pH. *L. iners*, on the other hand, despite belonging to the same genus, does not provide such unequivocal protection. The best-documented protective microbiota profile is associated with the dominance of *L. crispatus*, while other *Lactobacillus* configurations may have different biological value [7,8,18,19]. Thus, *Lactobacillus* dominance is not a uniform protective state: *L. crispatus* generally marks lower pH and stability, whereas *L. iners* often accompanies transitional or unstable communities.

Mechanistically, dysbiosis may facilitate HPV persistence in several ways. Higher pH and biofilm weaken the mucosal barrier. Anaerobic bacterial products induce local cytokine secretion and oxidative stress. Chronic inflammation may, over time, impair effective antiviral responses while simultaneously promoting angiogenesis, extracellular matrix remodeling, and immune escape. In such an environment, cells with impaired cycle control following E6/E7 expression are more likely to survive and undergo selection [2,5,6,16,17,20,21].

The most realistic clinical application of microbiota in cervical cancer does not involve replacing HPV testing. HPV testing, cytology, colposcopy, and histopathological diagnosis remain the cornerstones of management. However, a microbiota profile could, in the future, help identify which HPV-positive women are at greater risk of persistent infection and progression to CIN. Such an application requires prospective studies, a common sampling procedure, and control for confounding factors such as antibiotics, smoking, sexual activity, menstrual cycle status, menopause, and vaginal preparations [16,19,20,22].

Restoring an ecosystem dominated by *L. crispatus* makes biological sense. Both *L. crispatus*-containing preparations and vesicles derived from this species have been described, as well as pilot studies involving the *chen-01* strain [27,28,29,30]. This does not change the fact that probiotics are not an alternative to HPV vaccination, screening, and treatment of CIN. At this stage, they can be presented as a possible support requiring clinical validation, rather than as an anticancer therapy. The cited pilot study assessed HPV clearance or viral load and vaginal microbiota composition, not CIN regression or cancer outcomes [28]; microbiota-directed treatment therefore remains adjunctive and investigational.

HPV-based screening is increasingly combined with genotype- and age-informed risk stratification. Genotype distributions in CIN3 vary with age, and Polish data likewise show that HPV16, 31, and 52 account for most HSIL lesions; microbiota profiles, if validated, should therefore complement rather than replace established HPV risk variables [31,32].

## 5. Endometrial Cancer

Endometrial cancer is among the most frequently diagnosed gynecological cancers. Its incidence is rising as the population ages and as rates of obesity and metabolic disorders increase. In 2022, over 420,000 new cases were diagnosed worldwide, and over 97,000 deaths were reported [1]. The relationship between this cancer and the microbiota is less direct than that of cervical cancer, as there is no single etiological factor comparable to HPV.

The most likely outcome is a combination of several overlapping phenomena: the local uterine microbiota, the gut microbiota, the estrobolome, obesity, insulin resistance, and chronic inflammation. Each of these elements may be important, but none should be considered in isolation [10,11,12,33,34].

The uterine cavity is a niche with a low microbial population. This is important because in such samples, the line between a true biological signal and technical contamination can be thin. Collection of the sample through the vagina and cervix, the reagents used, the method of DNA extraction, and the timing of sample freezing can all influence the results. The old claim that the uterus is completely sterile is no longer convincing, but it would be equally risky to uncritically consider every detected taxon as a component of endometrial cancer biology [11,12].

Studies of patients with endometrial cancer have reported enrichment in potentially pro-inflammatory taxa, including *Porphyromonas*, *Atopobium*, and *Gardnerella*, and associations of specific microbiological signatures with disease, particularly in postmenopausal women [11,12,26]. In some cohorts, the presence or prevalence of specific bacterial species co-occurred with disease and may be important for developing risk assessment models, but these require validation.

**Figure 3:**
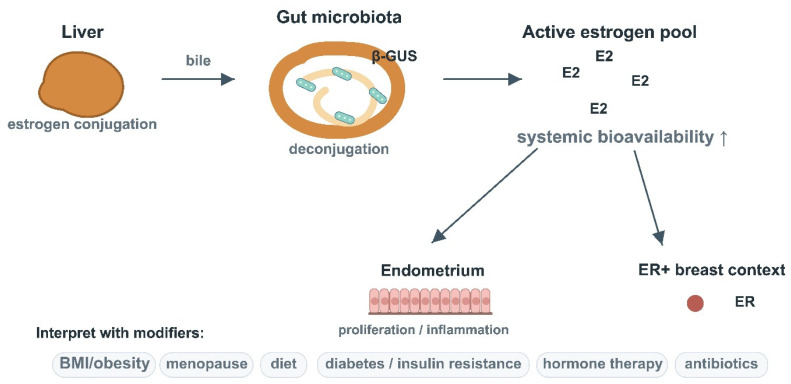
Gut–estrobolome–estrogen-sensitive tumor axis. Gut microbial β-glucuronidase activity may increase deconjugation and reabsorption of estrogens, thereby influencing systemic estrogen bioavailability. This axis is biologically relevant for endometrial cancer and selected hormone-dependent breast cancer contexts, but its interpretation requires integration with BMI, menopausal status, diet, medication use, metabolic disease, and hormonal exposure.

The strongest biological link between the intestine and endometrial cancer is the estrobolome, a set of gut microbiota genes encoding proteins that metabolize estrogens (Figure 3). Excessive estrogen exposure is a well-documented risk factor for endometrioid cancer. Gut microbiota, through beta-glucuronidase activity, may influence estrogen deconjugation and reabsorption [10]. This mechanism is particularly relevant for patients who have obesity, metabolic disorders, and ongoing low-level inflammation. However, it does not explain the entire biology of endometrial cancer, especially non-endometrioid tumors and tumors with distinct molecular profiles [10,12,33,34].

The therapeutic dimension of endometrial cancer primarily concerns the gut-immune axis and treatment complications. The gut microbiota may influence the response to checkpoint blockade, and interventions such as fecal microbiota transplantation (FMT), probiotics, or diet are being considered as future strategies for personalized treatment [35,36,37,38,39,40]. However, in clinical practice, this remains a research topic. Similarly, data on reducing radiotherapy toxicity by modulating the gut microbiome are promising but not yet standard practice [41].

At the clinical level, the microbiota in endometrial cancer is currently more of a translational hypothesis than a ready-made tool. One could envision future models including BMI, menopause, hormonal exposure, diabetes, tumor molecular profile, inflammatory markers, and microbiota data. Such a model would be more valuable than a single taxon [10,11,12,33]. Future studies should stratify or adjust for menopause, obesity, diabetes, antibiotics, hormonal therapy, bleeding, and sampling route. They should also test associations with molecular subtype, mismatch-repair deficiency, and immune contexture rather than extend the estrobolome model to all tumors [12,33,34].

## 6. Ovarian Cancer

Ovarian cancer poses the greatest interpretive challenge. It is often diagnosed late, encompasses several biologically distinct histotypes, and microbiota testing material most often comes from surgery or advanced disease. In 2022, over 320,000 new cases and over 206,000 deaths were reported [1]. About 70% of ovarian cancer cases are identified only after the disease has reached an advanced stage, and it is common for the cancer to relapse after treatment. These characteristics search for additional biomarkers, including microbiological ones, understandable, but it must be conducted with rigorous methodological control.

The ovary, fallopian tube, and peritoneum are environments with low microbial content, so the risk of technical error is real. In addition, antibiotics, prior procedures, chemotherapy, endometriosis, ascites, tumor necrosis, and the patient’s nutritional status can alter microbiota composition without reflecting carcinogenesis [42,43,44,45]. Thus, human signals are hypothesis-generating and may reflect sampling or treatment rather than tumor biology.

The study by Nenė et al., which assessed the association of cervicovaginal microbiome, BRCA1 status, and ovarian cancer risk, is interesting [42]. Its significance rests not in proving causality but in shifting the question: the microbiota may be an environmental component that modulates risk in genetically predisposed women. This is an important hypothesis, but it requires replication and prospective studies.

Experimental studies link gut microbiota to Hedgehog signaling, microbial metabolites, inflammation, epithelial–mesenchymal transition, and immune remodeling in ovarian cancer [43,44,45,46]. These putative mechanisms have not been validated as clinically actionable targets and currently support hypothesis generation only.

Diagnostically, assessing the microbiota composition does not replace CA125, HE4, ultrasound, or hereditary risk assessment. If used in practice, it will likely be a component of multiparameter models, along with BRCA1/2 status, histotype, imaging features, inflammatory markers, and tumor molecular profile [42,43,44,45,47]. This approach is more reliable than searching for a single bacterium that would distinguish early ovarian cancer from benign lesions.

Evidence from melanoma and other tumors links gut microbiota to PD-1/PD-L1 response, and FMT has overcome resistance in some melanoma patients [35,36,37,38]. These results cannot be transferred directly to ovarian cancer, which differs in immune responsiveness and mutational landscape and is further shaped by histotype, peritoneal spread, ascites, PARP inhibitors, chemotherapy, and antibiotics. Disease-specific trials are required.

## 7. Breast Cancer

Breast cancer is the most commonly diagnosed cancer among women. Its inclusion in this review is comparative, as it allows identification of mechanisms common to female oncology: the influence of the gut microbiota on estrogen metabolism, the importance of the intratumoral microbiome, and modulation of treatment response [1,9,13,14,15,23,24,48]. These comparisons are mechanistically informative but cannot substitute for evidence from gynecologic cancers, which differ in tissue biology, hormonal regulation, immune context, and treatment.

The estrobolome is the most relevant topic in breast cancer. The gut microbiota can influence the pool of active estrogens, thereby indirectly affecting estrogen-sensitive tissues. This mechanism is most significant in the context of hormone-dependent cancers, obesity, diet, and menopause [9,10]. However, this does not mean that modifying the gut microbiota alone can be considered a prevention or treatment for breast cancer.

**Figure 4:**
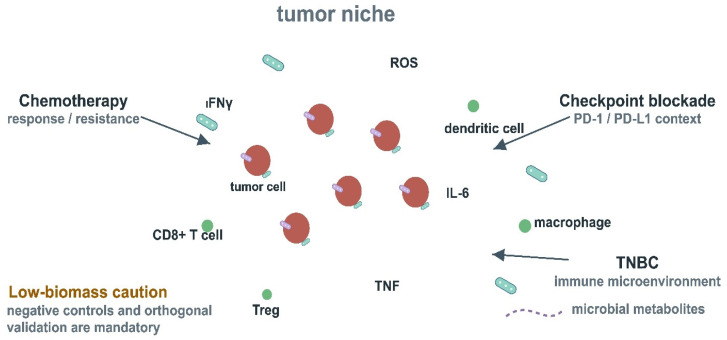
Intratumoral microbiome and tumor immune microenvironment. Intratumoral bacteria may interact with cancer cells, stromal cells, and immune infiltrates, shaping the inflammatory tone, macrophage and dendritic cell activity, CD8-positive T-cell function, and response to chemotherapy or immunotherapy. Because tumor tissue is often a low-biomass material, these findings require strict contamination control and independent validation.

The second direction is the intratumoral microbiome (Figure 4). Bacteria belonging to the *Proteobacteria*, *Firmicutes*, *Actinobacteria*, and *Bacteroidetes* groups have been detected in breast cancer tissue, and the microbiome composition may differ between biological tumor subtypes [13,23,48,49,50]. In some studies, the intratumoral microbiota has been associated with immune infiltration, inflammatory signals, and treatment response.

Research on the microbiome in triple-negative breast cancer is expanding rapidly. In TNBC, tumor and gut microbiota may co-shape the immune microenvironment and chemoimmunotherapy response [24,51]. Microbiota-linked epigenetic changes and metabolites such as butyrate are also under study [52,53], but neither is ready for clinical use.

In breast cancer, research shows that microbiota may play a role not only on mucosal surfaces but also within the tumor itself and in response to systemic treatment. It is important to note that the lower the sample biomass and the more complex the treatment, the greater the need for methodological control.

## 8. Biomarker and Therapeutic Significance

The clinical application of microbiota will likely involve building complex models rather than introducing a single microbiological test. In cervical cancer, a microbiota profile could support risk assessment in HPV-positive women. In endometrial cancer, microbiological data would need to be combined with BMI, menopause, metabolic status, and tumor molecular profile. In ovarian cancer, the diagnostic value of the intratumoral microbiome requires special caution, whereas in breast cancer, it could serve as a predictor of treatment response (Table 2) [10,11,12,16,23,24,33,42,43,44,45,53].

Microbiota-targeted interventions include probiotics, prebiotics, dietary modification, polyphenols, FMT, and VMT. Biologically, these interventions make sense: they can modulate the mucosal barrier, inflammation, metabolites, and immune response [27,28,29,30,39,40,41,54]. However, translating these observations into hard endpoints such as HPV clearance, CIN progression, response to immunotherapy, progression-free survival, or quality of life after radiotherapy remains a challenge.

Four levels of application should be separated: microbiota profiling as a research tool; microbial signatures within multiparameter risk models; prediction of treatment response; and microbiota-targeted therapy. Only the first two approach translation, chiefly in HPV-positive cervical disease; response prediction and intervention remain investigational.

Safety is critical. FMT and VMT require rigorous donor screening, pathogen surveillance, preparation standards, and adverse-event monitoring; probiotics also require strain-level characterization. Because oncology patients may be immunocompromised by treatment, surgery, malnutrition, or advanced disease, routine use without clear indications and clinically meaningful endpoints should not be considered risk-free.

## 9. Limitations of Current Research

**Figure 5:**
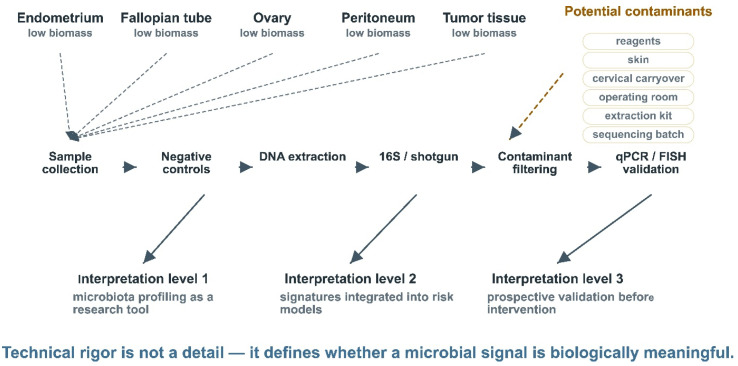
Methodological control in low-biomass microbiome studies. Microbiome studies of the endometrium, ovary, peritoneum, and tumor tissue are highly sensitive to contamination from reagents, skin, the cervical canal, and the surgical environment. Reliable interpretation requires standardized sampling, negative controls, transparent DNA extraction and sequencing protocols, contaminant filtering, prospective validation, and integration with clinical, histopathological, and molecular data.

The greatest limitation of microbiota research in female oncology is the heterogeneity of methods. There are differences in sampling sites, sample preservation methods, DNA extraction protocols, selection of the 16S rRNA region, sequencing depth, criteria for contaminant removal, and bioinformatics analysis methods (Figure 5). As a result, study results can be difficult to compare, even when they concern the same disease [11,12,13,14,15].

A second problem is low-biomass samples. Endometrium, fallopian tube, ovary, peritoneum, and tumor tissue contain low numbers of microorganisms compared to the intestine or vagina. In such conditions, reagent or environmental contamination may become the dominant signal. Therefore, each study should include negative controls, a description of the sample collection route, analysis of potential contaminants, and independent validation, preferably using an alternative method, e.g., qPCR, FISH, or shotgun metagenomics [13,14,15].

The third limitation concerns confounding factors. The microbiota is influenced by age, menopause, menstrual cycle phase, sexual activity, smoking, diet, BMI, diabetes, antibiotics, vaginal medications, menopausal hormone therapy, chemotherapy, radiotherapy, and surgery. Without controlling for these variables, it is easy to attribute disease to changes resulting from treatment, lifestyle, or the sampling procedure itself [2,3,12,16,41].

Study design is another limitation: cross-sectional comparisons cannot show whether dysbiosis precedes or follows cancer. A minimum standard should include standardized, preferably pretreatment sampling; documented route and relevant exposures; negative extraction and sequencing controls; contaminant filtering; longitudinal follow-up; and orthogonal validation by qPCR, FISH, culture, or shotgun metagenomics where appropriate.

## 10. Conclusions

Evidence strength is uneven: it is strongest for cervicovaginal microbiota in HPV persistence and cervical neoplasia; moderate and mechanistically plausible for gut-estrobolome effects in endometrioid and hormone-dependent breast cancer; and exploratory for ovarian cancer and many intratumoral applications.

The safest translational conclusion is that microbiota can enrich risk models, serve as adjunctive diagnostics, and support personalized treatment, but it should not currently be presented as a standalone clinical marker. Future studies must be prospective, multicenter, methodologically standardized, and linked to classical clinical, histopathological, and molecular data.

## Figures and Tables

**Table 1:** Most important observations regarding the microbiota in selected cancers in women.

Tumor	The Most Frequently Described Pattern	Possible Biological Interpretation	The Most Important Caveat
Cervical cancer	Decrease in the stable dominance of *L. crispatus*; increase in the proportion of *Sneathia*, *Prevotella*, *Porphyromonas*, *Anaerococcus*, and *Gardnerella*.	An environment with a higher pH and greater inflammatory potential may facilitate hrHPV persistence and CIN progression.	Strongest data, but prospective validation of risk stratification is still needed.
Endometrial cancer	Low-biota uterine microbiota; enrichment of pro-inflammatory taxa and the importance of the gut-estrogen axis in some studies.	Estrobolome, obesity, insulin resistance, and inflammation may co-create an environment conducive to endometrioid cancer.	High risk of contamination and strong influence of menopause, BMI, diabetes, and medications.
Ovarian cancer	Data on cervicovaginal, intestinal, and intratumoral microbiota reveal interesting correlations with BRCA1/2, inflammation, and metabolites.	The microbiota may modulate risk, immunity, EMT, metabolites, and treatment response.	Most exploratory area; no ready-made diagnostic biomarker
Breast cancer	Gut microbiota and the estrobolome; intratumoral microbiota associated with tumor phenotype and treatment response, especially in TNBC.	Possible influence on estrogen signaling, immune infiltration, and response to chemoimmunotherapy.	Comparative section; results should not be automatically extrapolated to gynecologic oncology.

**Table 2:** Possible clinical applications of microbiota and requirements before implementation.

Direction	Possible Use	What Needs to Be Refined	Clinical Readiness
Cervicovaginal Microbiota	Complementing the risk assessment for HPV-positive women with CIN.	Common collection protocols, control of antibiotic intake, smoking, sexual activity and cycle phase.	The most promising, but not routine.
Estrobolom	Risk models for endometrial and subtypes of breast cancer.	Monitoring BMI, menopause, diet, medications, menopausal hormone therapy and metabolic diseases.	Early translation.
Intratumoral microbiome	Prediction of response to treatment, especially immunotherapy and chemoimmunotherapy.	Contamination, low biomass, validation in independent cohorts and analysis with tumor microenvironment.	Research: rapid development.
Probiotics/Prebiotics/FMT/VMT	Modulation of the mucosal or intestinal environment; potential support for treatment.	Hard endpoints, safety, strain/donor selection, antibiotic and dietary control.	FMT/VMT for research purposes only; probiotics with no anticancer treatment status.

## Data Availability

No new data were created or analyzed in this study. Data sharing is not applicable to this article.
